# Acute Toxicities of Three Para-Phenylenediamine Quinones to Coho Salmon (*Oncorhynchus kisutch*) Juveniles and Embryonic Cells

**DOI:** 10.1007/s00128-025-04150-6

**Published:** 2025-11-30

**Authors:** Hiroyuki Mano, Akihiro Moriyama, Junko Hara, Rie Tai, Wataru Naito

**Affiliations:** 1 National Institute of Advanced Industrial Science and Technology (AIST), Research Institute of Science for Safety and Sustainability, 16-1 Onogawa, Tsukuba, Ibaraki 305-8569 Japan; 2 National Institute of Advanced Industrial Science and Technology (AIST), Integrated Research Center for Nature Positive Technology, 1-1-1 Higashi, Tsukuba, Ibaraki 305-8565 Japan; 3 National Institute of Advanced Industrial Science and Technology (AIST), Integrated Research Center for Nature Positive Technology, 16-1 Onogawa, Tsukuba, Ibaraki 305-8569 Japan

**Keywords:** Acute toxicity, Cytotoxicity, Ecotoxicity, Salmon, Tire road wear particles, PPDQ

## Abstract

The acute toxicities of para-phenylenediamine quinones (PPDQs) to salmonids at the whole-organism level remain unclear. Therefore, in this study, we investigated the acute toxicities of three PPDQs, 6PPDQ, 7PPDQ, and 8PPDQ in coho salmon (*Oncorhynchus kisutch*) juveniles and embryonic cells (CSE-119) using 24-h acute toxicity tests and cytotoxicity assays. The 24-h 50% effect concentration values of 6PPDQ and 7PPDQ for the juveniles were 41.5 ng/L (95% confidence interval: 21.6–61.4) and 5.39 μg/L (2.26–8.53), respectively, while 8PPDQ did not affect mortality at the tested concentration (60.6 μg/L). Further, the 24-h 50% effect concentration value of 6PPDQ for CSE-119 was 14.9 μg/L (13.0–16.7), but 7PPDQ and 8PPDQ did not sufficiently reduce CSE-119 cell viability even at concentration up to 500 μg/L, indicating marked differences in toxicity among the compounds. These findings indicate that, at both the whole-organism and cellular levels, 6PPDQ exhibited the highest toxicity among the three PPDQs, likely due to differences in chemical structure.

## Introduction

6PPD-quinone (2-anilino-5-[(4-methylpentan-2-yl)amino]cyclohexa-2,5-diene-1,4-dione) (6PPDQ), an oxidation product of 6PPD (N-(1,3-dimethylbutyl)-N′-phenyl-p-phenylenediamine), which is an antiozonant that is extensively used in vehicle tires worldwide, is known to exhibit high toxicity to coho salmon (*Oncorhynchus kisutch*) (Tian et al. 2021; Lo et al. 2023). In recent years, numerous studies have reported its adverse effects on aquatic organisms, including several species of the genus *Oncorhynchus* (Foldvik et al. 2024; Shankar et al. 2025).

Several substituted p-phenylenediamine (PPD) compounds, which are used as antiozonants and belong to the same chemical class as 6PPD, can be oxidized to quinone derivatives. Ecotoxicological data for some of these PPD-quinones (PPDQs), based on bioassays using luminescent bacteria and fish cell lines, have been reported (Wang et al. 2024; Harris et al. 2025). Specifically, cytotoxicity tests involving rainbow trout (*Oncorhynchus mykiss*) cells suggest that the toxicity of 7PPDQ in salmonid species is comparable to that of 6PPDQ (Harris et al. 2025). However, the acute toxicities of these compounds in salmonids at the whole-organism level have not been fully investigated.

Bioassays using fish cell lines represent a promising high-throughput method for chemical toxicity assessment and safety evaluation of alternative additives (Nguyen et al. 2024). Notably, rainbow trout cells and whole organisms show comparable sensitivities to certain chemical classes (Tanneberger et al. 2013; Natsch et al. 2018). Although embryonic cells derived from coho salmon are available for such assays, data on the correlation between cellular and whole-organism sensitivity for coho salmon, particularly to PPDQs, have not yet been reported.

In this study, we conducted 24-h acute toxicity tests using coho salmon individuals to evaluate the toxicities of three PPDQ compounds (6PPDQ, 7PPDQ, and 8PPDQ) to this species. Cytotoxicity assays using coho salmon embryonic cells were also performed to compare and assess the relationship between cellular and organismal sensitivities to these compounds.

## Materials and Methods

### Test Substances

The test compounds 6PPDQ, 7PPDQ, and 8PPDQ (96.9%, 98.0%, and 97.4% purity, respectively) were kindly provided by Bridgestone Corporation (Tokyo, Japan) and used as received without further purification.

### Test Organisms

Eyed eggs of coho salmon (Oncorhynchus kisutch) were obtained from the Inland Water Fisheries Research Center, Miyagi Prefectural Fisheries Technology Institute, Japan. The coho salmon population at this facility was established from eyed eggs originally obtained from Washington State, USA and introduced in 1987, and they were maintained through successive generations. The eggs were fertilized in November 2024 and initially reared in Atkins-type incubators at the Research Center. After health inspection on December 9, 2024, they were transferred to IDEA Consultants, Inc. (Yaizu, Shizuoka, Japan) for further incubation under the following conditions: flow rate, 7.7 L/min; average temperature, 9.5 °C; and pH, 7.69. Hatching began on December 25, 2024 (42 days post-fertilization), and feeding commenced on January 20, 2025 (68 days post-fertilization). Then, on February 13, 2025 (92 days post-fertilization), the resulting juveniles were transferred to 30-L glass tanks with flow-through conditions as follows: flow rate ≥ 0.5 L/min; water turnover, 24 volumes/day; and temperature, 10–12 °C. The fish density was maintained at ≤ 10 individuals/L (150–300 fish per tank), and a 16-h/8-h light–dark cycle was applied. On March 10, 2025 (117 days post-fertilization), the fish were transferred to the National Institute of Advanced Industrial Science and Technology (AIST), where they were reared in recirculating flow-through 2- and 4-L tanks prior to the acute toxicity tests. Specifically, the fish were acclimated in 2- or 4-L tanks, in which the fish density maintained at 10 fish/L and the water turnover rate was 24 volumes/day. The flow rates in the 2- and 4-L tanks were set at ≥ 33 and ≥ 67 mL/min, respectively, and for both systems, a 16-h/8-h light–dark cycle was applied, with the water temperature and dissolved oxygen (DO) level maintained at 13 °C and >70% saturation, respectively

The fish were fed commercial salmon feed (EX Masu LPS No.1 and No.2 EX Crumble, Nosan Corporation, Kanagawa, Japan). Prior to transfer to 30-L tanks, the feeding rate was set at approximately 3% of body weight per day. Thereafter, fish were fed at 70–80% of their satiation level. The food was provided daily until 24 h before the start of the toxicity test. The ages of the fish used in the 6PPDQ, 7PPDQ, and 8PPDQ toxicity tests were 134, 154, and 141 days post-fertilization, respectively, reflecting the sequential order of testing (6PPDQ testing was first, followed by 8PPDQ and then 7PPDQ). Their mean body lengths (mouth to tail base), and wet weights were 3.97 ± 0.64 cm and 0.77 ± 0.37 g (mean ± SD, N = 12), 3.90 ± 0.23 cm and 0.80 ± 0.18 g  (N = 10), and 4.1 ± 0.61 cm and 0.62 ± 0.12 g (N = 10), respectively.

### Test Cells

The coho salmon CSE-119 embryonic cell line (Lot: 06A009) used in this study was obtained from KAC Co., Inc., Kyoto, Japan. The cells were maintained at 19 ± 1 °C in Leibovitz’s L-15 medium (Wako, Japan) supplemented with 10% fetal bovine serum (FBS; Gibco, Waltham, MA, USA), 100 U/mL penicillin, and 100 μg/mL streptomycin (Wako, Osaka, Japan). The cells were maintained in tissue culture-treated flasks and subcultured after they reached approximately 80–90% confluence.

### Acute Toxicity Tests

The 6PPDQ and 7PPDQ exposure tests were conducted using a solvent control and five 6PPDQ and 7PPDQ concentrations. The exposure concentrations of 6PPDQ were 32, 63, 125, 250, and 500 ng/L, while those of 7PPDQ were set below 100 μg/L at 1.5, 3.3, 7.3, 16.1, and 35.4 μg/L based on a preliminary limit test using a 100-μg/L 7PPDQ solution that resulted in harmful effects. For 8PPDQ, a limit test was conducted using a solvent control and a single concentration at 100 μg/L, and given that no visible coloration was observed in the test solution at this 8PPDQ concentration, the 8PPDQ assay was performed at this concentration.

Two 5-L glass beakers each containing 4-L of the test solution per treatment were prepared for each PPDQ by adding stock solutions to the 4-L test medium at different concentrations under stirring at 550 rpm using a 45 × 8-mm magnetic stirrer (KSS-12; AS ONE, Osaka, Japan). Dechlorinated tap water filtered through activated carbon, with the water chemistry parameters as provided in Table S1, was aerated overnight and used to prepare the test solutions. Stock solutions of 6PPDQ, 7PPDQ, and 8PPD were prepared via dissolution in DMSO at concentrations of 5 mg/L, 500 mg/L, and 5 g/L, respectively. Additionally, DMSO was added to obtain a final solvent concentration of 0.01% (v/v) in all the test solutions, followed by stirring at 550 rpm for 3 min. Thereafter, four coho salmon juveniles were placed in one beaker and three were placed in another beaker for each treatment. In accordance with OECD Test Guideline No. 203, the loading of fish wet weight was maintained below 0.8 g/L (Tables S2–S4). Then, the exposure tests were then performed at 13 ± 1 °C under a 24-h dark/0-h light photoperiod, and at 1, 2, 4, 6, and 24 h, the number of dead individuals in each test was recorded. For all the test solutions, pH (AS800, AS ONE, Osaka, Japan), dissolved oxygen (DO) levels (FDO 380, AS ONE, Osaka, Japan), and water temperature (CT-320WP, CUSTOM, Tokyo, Japan) were measured at the start and end of each test.

### Cell Toxicity Test

CSE-119 cells were seeded into black 96-well cell culture plates (Nunclon Delta, Black Microwell SI, #137101) at 2.0 × 10^4^ cells per well by dispensing 100 μL of a CSE-119 cell suspension (2.0 × 10^5^ cells/mL) in each well. After pre-incubation for 24-h to allow for cell attachment, the culture medium was removed, and the test compound was added. First, the test compound was dissolved in DMSO to a concentration of 1 mg/mL, and the resulting solution was further diluted with L-15 medium containing 1% penicillin–streptomycin without FBS to obtain the exposure concentrations. The highest final concentration was 500 μg/L with 0.05% DMSO, and two-fold serial dilutions were further performed to reduce the concentration to 0.97 μg/L. Each concentration was tested in four replicates. Specifically, 100 μL of each test solution was added to each well and incubated for 24 h at 19 °C, after which the exposure solution was removed and the wells were washed with HBSS. Next, 100 μL of HBSS containing 10% Alamar Blue reagent (Invitrogen; Thermo Fisher Scientific Inc.) was added to each well, followed incubation for 1 h. Fluorescence measurements were performed at excitation and emission wavelengths of 530 and 595 nm, respectively, using the Infinite 200 PRO Nano + plate reader (Tecan Group Ltd., Zürich, Switzerland). Then, cell viability was then determined and expressed relative to the fluorescence of cells exposed to the solvent control (0.05% DMSO), considered to exhibit 100% fluorescence.

### Chemical Analysis

For chemical analysis, 1 mL of the test solution in each acute toxicity test beaker was sampled using a micropipette with 1000-μL low retention and an ultraclear filtered tip (QSP 1000, Thermo Fisher Scientific Inc.) to determine the test chemical concentrations before pH and DO measurements at the start and end of the tests. The samples thus collected were stored at 4 °C and subsequently analyzed for the PPDQs via liquid chromatography-mass spectrometry (LCMS-8060, Shimadzu Co. Ltd.). Details regarding the analytical method are provided in Text S1. For each beaker, the geometric mean of the measured concentrations at the start and end of the test was calculated. The arithmetic mean of these geometric means from the two beakers in each concentration group was then used to derive the toxicity values.

### Statistical Analysis

The proportions of individuals that survived in each acute toxicity tests and the obtained cell viabilities from the cytotoxicity tests were statistically analyzed using R software version 4.4.1 to determine the 24-h 50% lethal concentrations (LC_50_) and 50% effect concentrations (EC_50_) of the test chemicals. Corresponding 95% confidence intervals were determined using the drc package in R (Ritz et al. 2015). Data on survival and cell viability were fitted to two-parameter log-logistic (LL.2) models with binomial error distributions and four-parameter log-logistic (LL.4) models with gaussian error distributions using the drm function.

## Results and Discussion

### Acute Toxicities of the Three PPDQs

**Fig. 1:**
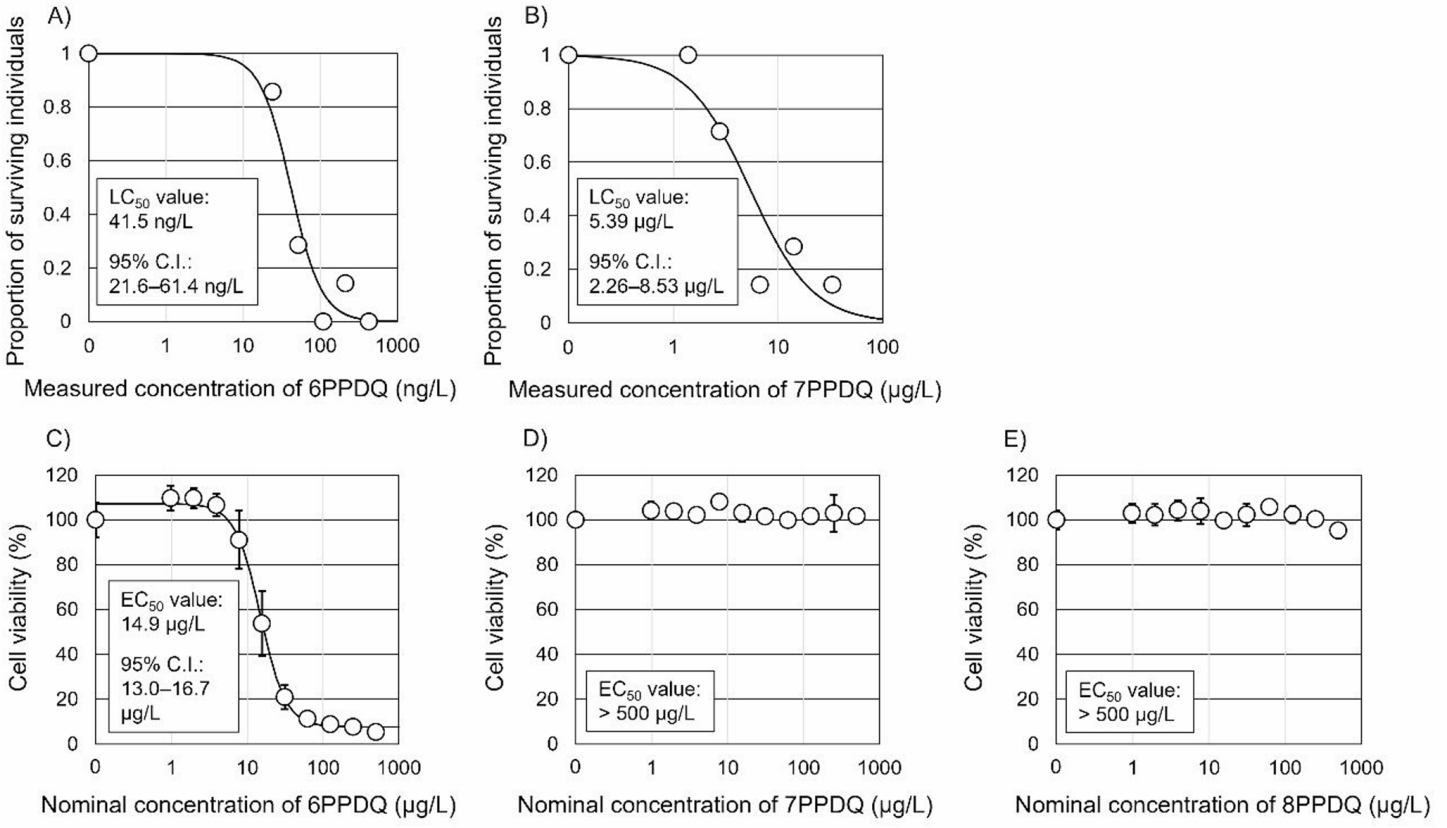
Responses of coho-salmon individuals to different **A** 6PPDQ (ng/L) and **B** 7PPDQ (μg/L) concentrations. Responses of coho salmon embryonic cells to **C** 6PPDQ (μg/L), **D** 7PPDQ (μg/L), and **E** 8PPDQ (μg/L). LC_50_ values and the 95% confidence intervals for 6PPDQ and 7PPDQ toxicities to coho salmon following exposure were estimated by fitting observation data to two-parameter log-logistic models with binomial error distributions. The EC_50_ value and its 95% confidence interval for 6PPDQ-induced declines in cell viability were estimated using a four-parameter log-logistic model with a Gaussian error distribution

The impacts of three PPDQs on the survival of coho salmon were investigated. Summaries of the pH, DO levels, water temperature, measured PPDQs concentrations, and fates of test individuals are provided in Tables S5–S14. Figure 1A and B show the dose response curves of 6PPDQ and 7PPDQ, respectively, and Table S15 provides detailed results of statistical analyses. The LC_50_ values of the three PPDQs decreased according to the following trend: 8PPDQ (>60.6 μg/L) >7PPDQ (5.39 μg/L, 2.26–8.53 μg/L [estimated LC_50_, 95% confidence limits]) >6PPDQ (41.5 ng/L, 21.6–61.4 ng/L). This observed high toxicity of 6PPDQ to the test animals is consistent with that previously reported by Lo et al. (2023) (41 ng/L) based on experiments in which coho salmon juveniles were exposed to 6PPDQ for 24 h under static conditions. To the best of our knowledge, this study is the first to report the acute toxicities of 7PPDQ and 8PPDQ in coho salmon.

The ecotoxicities of the three PPDQs investigated in this study to other species have been previously reported (Wang et al. 2024). Predictions of their ecotoxicities to salmonid species have also been reported (Harris et al. 2025). Wang et al. (2024) conducted acute toxicity tests for 6PPDQ, 7PPDQ, and 8PPDQ using the aquatic bacterium *Vibrio fischeri* and reported EC_50_ values of 15.56 ± 9.85, 14.91 ± 8.20, and 11.05 ± 5.81 mg/L, respectively. Based on these findings, they concluded that 7PPDQ and 8PPDQ are more toxic to coho salmon than 6PPDQ. However, in the present study, the toxicity of 6PPDQ to coho salmon was significantly greater than those of 7PPDQ and 8PPDQ, implying a different toxicity ranking relative to that observed for *V. fischeri*. Based on the estimated toxicity values, the differences in toxicity among 6PPDQ, 7PPDQ, and 8PPDQ to *V. fischeri* appeared to be minimal. In contrast, the observed toxicity values of these compounds to coho salmon in this study were significantly different.

Based on the results of the 6PPDQ and 7PPDQ cytotoxicity tests using the RTgill-W1gill cell line of rainbow trout (*Oncorhynchus mykiss*), Harris et al. (2025) suggested that the toxicity of 7PPDQ in salmonid species may be similar to that of 6PPDQ. Conversely, the present study showed a lower coho salmon toxicity for 7PPDQ than 6PPDQ, indicating that the difference in coho salmon toxicity between 6 and 7PPDQ is more pronounced than that between the values predicted based solely on exposure to rainbow trout cells. Regardless, the mechanism underlying this difference in toxicity between the compounds remains unclear and needs to be clarified.

In the 6PPDQ acute toxicity tests, decreases in 6PDQ concentration (the three lower exposure levels with nominal concentrations 32, 63, and 125 ng/L (0.107, 0.211, 0.419 nM)) tended to be smaller in beakers containing three test individuals than in those containing four individuals (Table S9). The underlying mechanism of this concentration decline was not elucidated in the present study, but it is possible that the number of exposed individuals in the beaker influenced the rate of 6PPDQ dissipation.

### Cytotoxicities of the Three PPDQs to CSE-119 Cells

The cytotoxicities of the three PPDQs to CSE-119 cells were assessed via Alamar Blue assays after 24 h of exposure. As shown in Fig. 1C, only 6PPDQ induced a clear concentration-dependent decrease in metabolic activity in the cells (14.9 μg/L, 13.0–16.7 μg/L [estimated EC_50_, 95% confidence limits]). Detailed statistical analysis results are provided in Tables S16. Conversely, even at the highest concentration (500 μg/L), 7PPDQ and 8PPDQ did not sufficiently reduce CSE-119 cell viability to allow EC_50_ estimation (Fig. 1C and E). Cell viability remained above 90% for these two PPDQs across all tested exposure concentrations. This observation indicates that CSE-119 cells are selectively sensitive to 6PPDQ. This selective cytotoxicity of 6PPDQ is consistent with the findings of Greer et al. (2023), who reported that 6PPDQ exerts metabolic and cytotoxic effects on CSE-119 cells. However, the EC_50_ value (for metabolic activity) of 6PPDQ was somewhat higher than the previously reported value (14.9 μg/L vs. 7.8 μg/L), possibly owing to slight differences in experimental conditions, such as differences in medium composition, chemical handling, and exposure timing.

Additionally, the higher toxicity of 6PPDQ compared with that of the other PPDQs may be related to its quinone structure and redox-cycling activity. Mahoney et al. (2022) reported that 6PPDQ increases oxygen consumption and induces mitochondrial uncoupling in primary rainbow trout gill cells, suggesting mitochondrial dysfunction as a plausible mechanism of 6PPDQ toxicity. Although mitochondrial activity was not directly measured in this study, the concentration-dependent decline in metabolic activity observed in CSE-119 cells is consistent with such a mechanism.

The absence of toxicity for 7PPDQ and 8PPDQ to CSE-119 cells suggested that structural differences in the side chains of PPDQs possibly determine their toxicities. Thus, further studies are required to investigate whether these analogs are less reactive, less bioavailable, or are associated with different metabolic pathways. Additionally, these findings support the utility of CSE-119 cells for in vitro toxicity evaluation of transformation products derived from tire-associated chemicals. The observed selective toxicity of 6PPDQ highlights the need to consider compound-specific effects and potential toxicity mechanisms when assessing environmental risk.

### Relationship Between Acute Toxicity and the Cytotoxicity Values of PPDQs

Comparison of whole-organism survival and cell viability after exposure to the three PPDQs indicated higher organism-level sensitivities than cellular-level sensitivities for 6PPDQ and 7PPDQ. Toxic effects of 6PPDQ observed at the cellular level are likely to manifest at the whole-organism level, suggesting that embryonic cell assays could serve as a screening tool to identify sites with elevated environmental concentrations of 6PPDQ. In contrast, the lack of cellular responses to 7PPDQ indicates limited applicability for such screening. Moreover, the organism- and cellular-level sensitivities to 6PPDQ were greater than those to 7PPDQ and 8PPDQ. Notably, in the acute toxicity test, sensitivity to 6PPDQ was approximately 130-fold and >1,460-fold higher than sensitivities to 7PPDQ and 8PPDQ, respectively. Assuming that these sensitivity ratios apply similarly to sensitivity estimates based on cell assays, the estimated sensitivities of coho salmon embryonic cells to 7PPDQ and 8PPDQ would be approximately 1.94 and >21.8 mg/L, respectively. These estimated concentrations exceed the upper limits of the concentration ranges of these PPDQs used in the cell assays (500 µg/L). Given the high concentrations required, 7PPDQ and 8PPDQ may not completely dissolve in the culture medium, making it difficult to accurately evaluate differences in cellular sensitivity to these compounds.

A limitation of this study is that only embryonic cells were used. As metabolic activity varies among tissues, sensitivity may differ. Given that fish primarily absorb chemicals through the gills, gill-derived cells may be more sensitive (Mahoney et al. 2022). Future studies should evaluate such cells to clarify inter-tissue and organism–cell sensitivity differences.

Taken together, the findings of this study revealed that the acute toxicity and cytotoxicity of 6PPDQ, 7PPDQ, and 8PPDQ to coho salmon differed. To clarify these differences and elucidate the underlying mechanisms, further investigations focusing on the differences between these PPDQs in terms of chemical structures and properties are crucial.
